# The Breast Cancer-Associated Glycoforms of MUC1, MUC1-Tn and sialyl-Tn, Are Expressed in *COSMC* Wild-Type Cells and Bind the C-Type Lectin MGL

**DOI:** 10.1371/journal.pone.0125994

**Published:** 2015-05-07

**Authors:** Richard Beatson, Gjertrud Maurstad, Gianfranco Picco, Appitha Arulappu, Julia Coleman, Hans H. Wandell, Henrik Clausen, Ulla Mandel, Joyce Taylor-Papadimitriou, Marit Sletmoen, Joy M. Burchell

**Affiliations:** 1 Breast Cancer Biology, King’s College London, Guy’s Hospital, London, SE1 9RT, United Kingdom; 2 Department of Physics, Norwegian University of Science and Technology, 7491, Trondheim, Norway; 3 Copenhagen Center for Glycomics, University of Copenhagen, Copenhagen, DK-2200, Denmark; University of Liverpool, UNITED KINGDOM

## Abstract

Aberrant glycosylation occurs in the majority of human cancers and changes in mucin-type O-glycosylation are key events that play a role in the induction of invasion and metastases. These changes generate novel cancer-specific glyco-antigens that can interact with cells of the immune system through carbohydrate binding lectins. Two glyco-epitopes that are found expressed by many carcinomas are Tn (GalNAc-Ser/Thr) and STn (NeuAc**α**2,6GalNAc-Ser/Thr). These glycans can be carried on many mucin-type glycoproteins including MUC1. We show that the majority of breast cancers carry Tn within the same cell and in close proximity to extended glycan T (Gal**β**1,3GalNAc) the addition of Gal to the GalNAc being catalysed by the T synthase. The presence of active T synthase suggests that loss of the private chaperone for T synthase, COSMC, does not explain the expression of Tn and STn in breast cancer cells. We show that MUC1 carrying both Tn or STn can bind to the C-type lectin MGL and using atomic force microscopy show that they bind to MGL with a similar deadadhesion force. Tumour associated STn is associated with poor prognosis and resistance to chemotherapy in breast carcinomas, inhibition of DC maturation, DC apoptosis and inhibition of NK activity. As engagement of MGL in the absence of TLR triggering may lead to anergy, the binding of MUC1-STn to MGL may be in part responsible for some of the characteristics of STn expressing tumours.

## Introduction

Glycosylation is one of the most widely found and complex post-translational modifications, and the glycome encompasses a vast and extensive repertoire of sugars covalently linked to proteins, glycolipids or proteoglycans. The mammalian glycome is estimated to contain thousands of different glycan structures, vastly expanding the diversity of the proteome, and is involved in key biological processes. Nearly all proteins that are expressed on the cell membrane, or are secreted, carry glycans and these are involved in cell adhesion, recognition, molecular trafficking, clearance and signalling [1]. Indeed, the recognition of specific carbohydrate chains (glycans) by carbohydrate-binding proteins (lectins) is an important regulatory mechanism of immune physiology in both health and disease [2].

Aberrant glycosylation occurs in the majority of human cancers, and changes in mucin-type O-glycosylation are key events that play a role in the induction of invasion and metastases [3–5], and generates novel cancer-specific glyco-antigens which can interact with cells of the immune system [6,7]. Mucin-type O-linked glycosylation of proteins is one of the most diverse forms of glycosylation because it involves 50–100 distinct genes, including up to 20 polypeptide GalNAc-transferases that control where the O-glycans are attached. In this type of O-linked glycosylation the first sugar added is N-acetylgalactosamine (GalNAc) and the polypeptide GalNAc-transferases catalyse the addition of this sugar to specific threonines and serines in the polypeptide chain [8]. In many tissues, including the mammary gland, the addition of a galactose to the initiating GalNAc forms the core 1 or T antigen. Only one enzyme is known to catalyse this reaction the core 1 **β**3galactosyltransferase, also known as T synthase. The activity of T synthase is totally dependent upon a private molecular chaperone known as Cosmc [9], which is located in the endoplasmic reticulum and prevents aggregation and degradation of T synthase [10].

**Fig 1:**
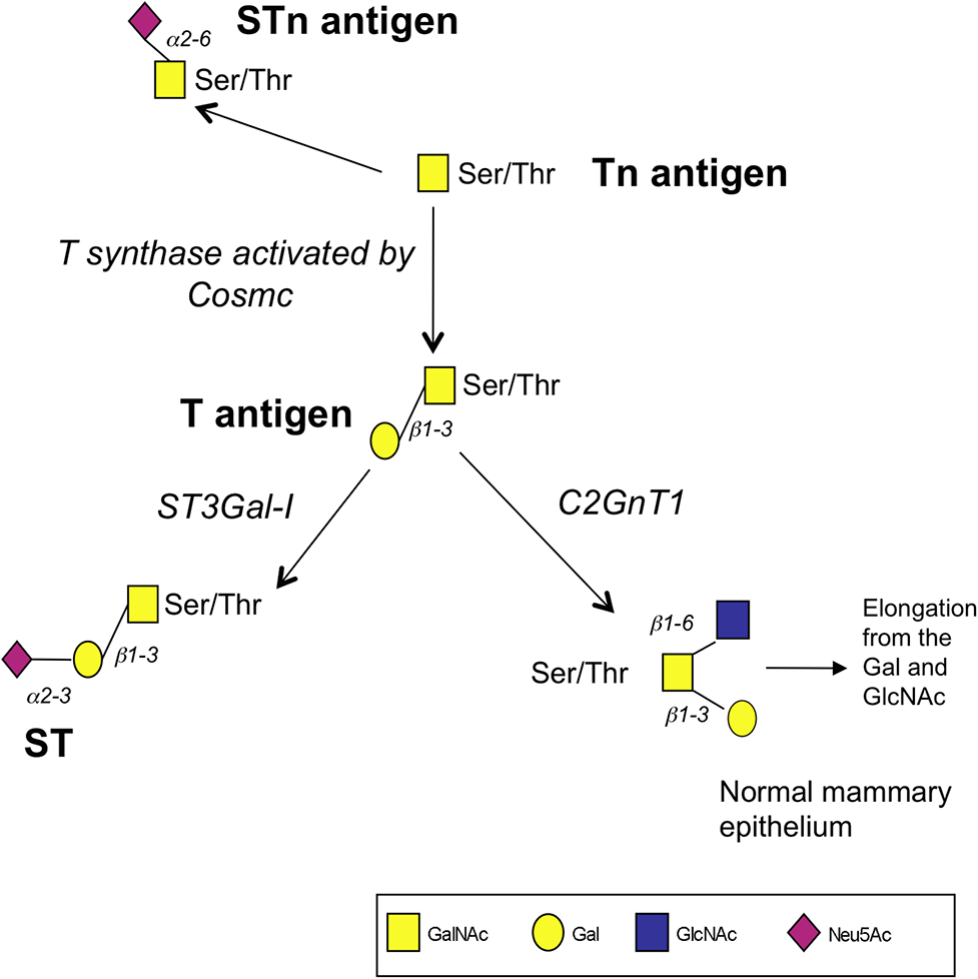
Schematic representation of the early stages of mucin-type O-linked glycosylation. After the addition of the first sugar (GalNAc) to serine or threonine the core 1 or T antigen is synthesized by the action of T synthase which requires the activity of COSMC for its action. Core1 can be further extended. If COSMC is inactive, glycosylation stops at Tn.

In the normal mammary gland the T glycans are extended further by the addition of *N*-acetylglucosamine (GlcNAc) to the GalNAc of core 1, forming a core 2 branch that can be further elongated (see Fig 1). However, in the change to malignancy in the breast, truncated O-linked glycans are often observed: unbranched glycans such as Tn (GalNAc), sialylated Tn (STn, NeuAc**α**2,6GalNAc), core 1 or T (Gal**β**1,3GalNAc) and sialylated T (ST, NeuAc**α**2,3Gal**β**1,3GalNAc) antigens are found attached to *O*-Ser/Thr, with the core 2 structures being absent or reduced (see Fig 1). The tumour-associated Tn antigen is expressed by more than 90% of breast cancers [11] while STn is found in 20–25% [12]. The T and ST glycans are expressed by the majority of breast cancers [13].

A number of mechanisms have been shown to be responsible for aberrant mucin-type glycosylation. In breast cancer, over-expression of the ST3Gal-I sialyltransferase results in the expression of ST [13] and the expression of STn is due to the turning on of the transcription of another sialyltransferase, ST6GalNAc-I [14]. However, it is not clear why the Tn glycan is so widely expressed although a number of mechanisms have been reported. These include mutations in the gene encoding COSMC resulting in loss of T synthase activity [15], hypermethylation of *COSMC* in pancreatic cancer [16] and relocation of polypeptide GalNAc transferases to the ER [17]. In breast cancer, the T and ST glycans are expressed together with the Tn glycan, suggesting that loss of COSMC function is not playing a major role in the expression of Tn. To examine how the core 1 based glycans are co-expressed with the Tn glycan we have focused on analysing glycoforms of the MUC1 mucin glycoprotein which is widely expressed in breast cancer and where a single molecule carries multiple glycans thus allowing evaluation of their juxtaposition.

Interactions of C-type lectins with the novel O-glycan-based antigens expressed in cancer can induce phenotypic changes in the lectin expressing cells. C-type lectin receptors bind specific carbohydrate ligands and stimulate uptake of antigen and secretion of cytokines such as interferons and interleukins, allowing this arm of the innate immune system to act as a first line of defence against pathogens [6]. However, antigens internalised through these C-type lectins can also be processed for presentation to T cells [18]. Although it was originally thought that C-type lectins acted exclusively to distinguish self from non-self, it is now clear that many self-glycans can be recognised. However, interactions in the absence of a danger signal may result in an anergic response. The C-type lectin MGL is found on antigen presenting cells, particularly macrophages and dendritic cells. Previous work has shown that MUC1-Tn peptides can bind to the C-type lectin MGL that is expresssed on monocyte derived immature dendritic cells [18,19], resulting in internationalisation and loading into MHC class I and II pathways [18].

Here we show that not only can MUC1-Tn interact with MGL, but also the sialyated derivative MUC1-STn can bind this lectin. The interaction with the two glycoforms is of a similar affinity as demonstrated by atomic force microscopy (AFM). This is of importance because although many carcinomas express MUC1 carrying Tn, this is rarely found at the surface of the cell [20]. Indeed, although the vast majority of breast cancers stain for Tn, this is only found at the cell surface in small amounts. In contrast, 20–25% of breast cancer are positive for STn and it is found on the cell surface [12], permitting interaction with MGL expressing cells.

## Materials and Methods

### MGL and Tn-, STn-, and ST-MUC1

Human recombinant MGL (accession number Q8IUN9) expressed in the mouse myeloma cell line NSO-derived, Gln61-His316, with an N-terminal 6-His tag was purchased from R and D Systems (Cat number 4888-CL-050).

MUC1-ST was produced as described in Backstrom et al [21]. MUC1-Tn and MUC1-STn were produced by transfecting CHO-ldlD cells [22], or wild-type CHO cells, previously tranfected with ST6GalNAc-I, with a human MUC1 (16 tandem repeat) mouse IgG2a Fc fusion construct as described in Backstrom et al [21]. MUC1 expressing clones were cultured in serum free medium in bioreactors as described in Noll et al [23], with the addition of 1mM exogenous GalNAc. The supernatant was filtered, concentrated (using a 100kD cut-off) and rebuffered in 200 mM Tris/HCl, pH 7.2, before 6mg was diluted to a concentration of 0.6mg/ml with 50mM Tris/HCl, pH 8.0, 0.05% Tween 20, 1mM CaCl_2_. To remove the Fc region, 500U of enterokinase (EK Max, Life Technologies) was added and the mixture was incubated at 37°C overnight, followed by a further overnight treatment with EK. The efficiency of cleavage was determined by silver staining. MUC1-Tn and MUC1-STn were purified by affinity chromatography using the human MUC1-Tn/STn specific 5E5 antibody [24] and the eluted fractions tested in a 5E5 sandwich ELISA to determine those containing MUC1-Tn or MUC1-STn. SDS PAGE was then performed followed by silver staining and Western blotting using HMFG1 (anti human MUC1 antibody) and 5E5. The eluted fractions were dialysed against PBS (3 x 5L DW) and the concentration and purity was confirmed by amino acid analysis (Alta Biosciences, Birmingham, UK).

### Chemoenzymatic synthesis of glycopeptides

Glycopeptides based on 3 tandem repeats of MUC1 carrying glycans on 9 of the 15 potential O-linked glycosylation sites were produced as described in Sorenson et al [11] and Tarp et al [24].

### Biotinylation and Digoxigenylation

Biotinylation of glycoproteins and antibodies was achieved by incubating each mg of product in 0.1M sodium carbonate buffer, with 60μg of biotinamidocaproate N-hydroxysuccinimide ester (Sigma) for 4 hours at room temperature. Free biotin was removed by dialysing overnight at 4°C against excess phosphate buffered saline containing 0.1% azide.

Digoxigenylation of antibodies was achieved by incubating each mg of antibody in PBS, with 320μg of Digoxigenin-3-0-succinyl-ε-aminocaproic acid-Nhydroxy-succinimide ester (Sigma) for 2 hours at room temperature followed by overnight dialysis at 4°C against excess phosphate buffered saline containing 0.1% azide.

### Cell lines

All cell lines used have been previously published and are referenced. K562 cells and K562 MGL-transfected cell line (KG11 [25]), a generous gift from Professor Tatsura Irimura, were cultured in RPMI 1640 (Lonza) supplemented with 100 units/mL penicillin, 100 μg/mL streptomycin, 2 mmol/L L-glutamine and 10% heat-inactivated FCS (all Life Technologies), 5% CO_2_ at 37°C. KG11 cells were cultured under selection with 400ug/ml G418 (Sigma) as described in Suzuki et al [25]. T47D-STn and T47D ‘SimpleCells’ were cultured in DMEM supplemented with 100 units/mL penicillin, 100 μg/mL streptomycin, 2 mmol/L L-glutamine and 10% heat-inactivated FCS (all Life Technologies) 5% CO_2_ at 37°C. T47D-STn cells were cultured under selection with 300ug/ml Hygromycin B as described in Sewell et al [14].

### Monocyte derived dendritic cell differentiation and maturation

Leukocytes cones were purchased from the National Blood Transfusion Service (NBTS, Tooting, UK) before being centrifuged on a Ficoll gradient (Ficoll-Paque PREMIUM, GE Healthcare) at 400G. After centrifugation CD14+ cells were isolated from PBMCs using CD14 microbeads (Miltenyi Biotech) and the MACS system, according to the manufacturers instructions. Purity was assessed at >95% by staining with an anti-human CD14 antibody.

The cells were plated at a concentration of 1x10^6^/ml in AIM V medium (Lonza) along with 1500U/ml recombinant human IL4 (Bio-Techne) and 400U/ml recombinant human GMCSF (Bio-Techne). The cells were incubated at 37C, 5% CO_2_ for 6 days to fully differentiate, before being characterised as immature DCs via phenotypic flow cytometric analysis (Epics XL, Beckman Coulter or FACSCalibur, BD Biosciences plus WinMDI or Cellquest software). DCs were matured using 1ug/ml LPS, with successful maturation being assessed by CD83 expression and the upregulation of CD80, CD86, CD40, CCR7 and HLA-DR as seen by flow cytometry.

### Phenotypic analysis

Cell phenotype staining was performed using the following antibodies: mouse anti-human MGL (HML clone, MoAb MLD-1 [25]; a generous gift from Professor Tatsura Irimura), mouse anti-human MUC1 (HMFG2), mouse anti-STn (TKH2,) followed by FITC-conjugated goat anti-mouse IgG (H+L; DAKO). MoAbs directly conjugated with FITC or phycoerythrin were also used (all from Beckman Coulter unless stated): IgG_1_-PE or FITC as isotype controls, anti-MGL (Biolegend; clone H037G3), anti-CD1a, anti-CD83, anti-CD80, anti-CD86 (all PE), anti-CD14, anti-HLA-DR, anti-CCR7, anti-CD40 (all FITC). Cells were suspended in PBS + 0.5% BSA (2 × 10^5^ cells/100 μL/sample) and incubated with MoAbs according to the manufacturer's instructions. At least 1 × 10^4^ events were evaluated using either Epics XL, (Beckman Coulter) or FACSCalibur (BD Biosciences) flow cytometers. Analysis was performed using either WinMDI or Cellquest software.

### Binding of MUC1 glycoproteins and glycopeptides to MGL expressing cells

Biotinylated glycopeptides or glycoproteins were bound for 2h to FITC streptavidin beads (Life Technologies) at 4°C in either calcium buffer (PBS + 0.5% BSA + 5mM Ca^2+^) or EDTA buffer (PBS + 0.5% BSA + 5mM EDTA) using 10ug beads and 5ug glycopeptide or glycoprotein/ml. After incubation the beads were spun at 4°C for 15 mins before being resuspended in appropriate buffer and spun again. 0.2x10^6^ cells (lines or primary cells) were plated and washed once in relevant buffer. 50μl of beads / glycoprotein/peptide was then added before being incubated for 2h on a rocking platform. The cells were washed twice in relevant buffer before being analysed by flow cytometry.

### Immobilization of MGL and MUC1 on mica surfaces and AFM tips

**Fig 2:**
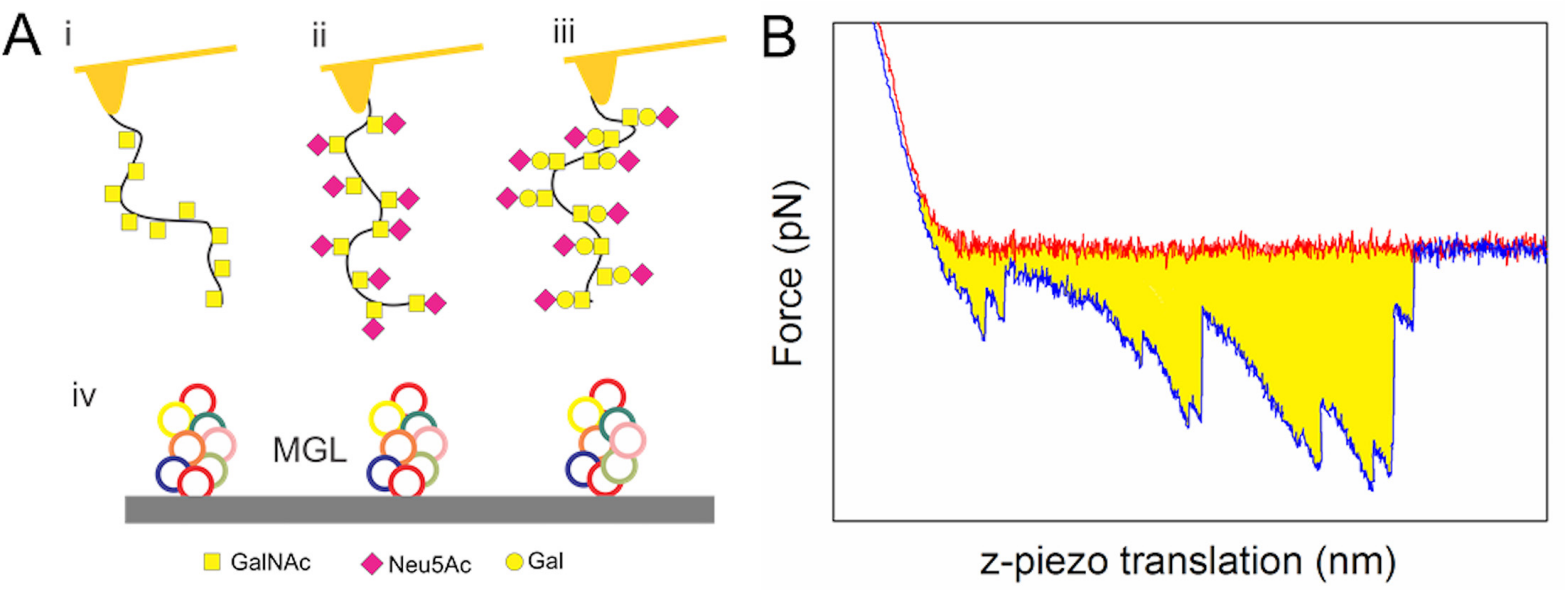
Schematic illustration of the method employed for determination of MUC1—MGL interactions by forced dissociation between immobilized MGL on a mica surface and MUC1 covalently attached to the AFM tip. A: Schematic illustration of AFM tips functionalized with MUC1-Tn (i), STn (ii) or ST (iii) and mica slides functionalised with MGL (iv). AFM tips and freshly cleaved mica was cleaned, silanised and functionalised as described in the text to give surfaces covered with covalently bound molecules. B: AFM force–distance curve obtained for a MUC1 functionalized AFM tip moved towards (red line) and away (blue) from the MGL functionalized mica surface. The work performed on the molecular system (Eq (1)) corresponds to the yellow area in the graph, where the baseline is determined from the approach data at large separation from the surface.

A schematic representation of the functionalized sample surface and AFM tips used in the present study is shown in Fig 2A. The functionalized surfaces were obtained using the following procedure: MGL was covalently linked to freshly cleaved mica (mineral muscovite) surfaces and purified recombinant MUC1 (Tn- STn- and ST-) to the AFM tips following a previously published protocol. [26,27 **]**. In brief, freshly cleaved mica was cleaned in 1:1 v/v concentrated HCl:MeOH (30 min), rinsed in MQ-water and silanized (freshly prepared 1% (v/v) solution of trimethoxysilylpropyl-diethylenetriamine in 1 mM acetic acid, for 20 min. at room temperature and rinsed in deionised-water). The amine-silanized layer was transformed to an aldehyde layer by incubating with glutaraldehyde (12.5% (v/v) in MQ-water, for 14 hours, rinsed in MQ-water and dried under N_2_). MGL was immobilized to the surface by conjugating the primary amine groups to the aldehyde groups of the immobilized cross linking agent (0.5 mg/ml MGL lectin in 100 mM Hepes buffer pH 7.2 containing 1mM CaCl_2_ and 1mM MnCl_2_, overnight, room temperature). MUC1 (Tn- ST- and STn-) were covalently anchored to separate AFM tips (Veeco OTR4-10 silicon nitride, nominal spring constant k = 0.02 N/m, silanized as the mica substrate) using EDC (1-(3-dimethylaminopropyl)-3-ethylcarbodiimide hydrochloride). The density of MUC1 on the AFM tip was controlled by selecting the MUC1 and EDC concentration. A high density was obtained by using the following conditions during the AFM tip functionalization reaction: 0.3 mg/ml MUC1 in 50 mM boric acid, pH 5.8, 10 mg/ml EDC. A lower density was obtained by reducing the concentration of both MUC1 and EDC to the following concentrations: 0.1 mg/ml MUC1 in 50 mM boric acid, pH 5.8, 1 mg/ml EDC. The incubation duration of the AFM tip was 1 hour.

### Determination of MGL—MUC1 forced unbinding

Force-distance curves obtained using AFM tips with high density of the immobilised MUC1 molecules were collected using the AFM ForceRobot 300 system (JPK instruments AG, Berlin, Germany), equipped with a JPK precision mapping stage and a liquid cell. The force-distance curves obtained using AFM tips with low density of the immobilized MUC1 molecules were collected using a Digital Instrument Multimode IIIa AFM equipped with a PicoForce controller (Santa Barbara, CA). The experimental approach was identical for both instrument set-ups. The data were collected in liquid (aqueous 100mM HEPES buffer pH 7.2 containing 1 mM CaCl_2_ and 1 mM MnCl_2_) at room temperature. Prior to each series of measurements the spring constant of the cantilever was calibrated using the method of thermal fluctuation [28] using the instrument software. For each MUC1—MGL sample investigated, forced unbinding data was collected using a z-piezo translation velocity of 0.2, 0.5, 1 and 2 μm/s. When using the AFM ForceRobot 300 system data series were obtained by performing 10 trials in 36 or 64 predetermined locations (6× 6 or 8× 8) evenly distributed across an area of 10×10 μm^2^ on the sample surface. Such data series were also collected upon addition of Tn-Ser, STn-Ser (Sussex Research, Ottawa, Canada) or GalNAc (Sigma) to the buffer solution contained in the liquid cell at concentration of 0.02, 0.5 or 1 mg/ml.

### Analyses of MGL—MUC1 forced unbinding

The data were analysed using a previously reported approach [29]. In brief, the data were analysed based on quantitative estimates of the work, W, performed by the applied force during the unbinding process. For repeat i in the experimental series at a given experimental condition the work W_i_ was estimated as follows:

$$W_{i}=\int_{z_{start}}^{z_{end}}F_{i}\left(z\right)dz$$ (1)

where z_start_ and z_end_ are the start and end of the integration of the force curve at retraction, F_i_(z), relative to the baseline for the experiment i. The z_start_ was selected as the contact point and z_end_ as the last unbinding event in each force curve (see Fig 2B). The baseline was selected based on the retract curve.

The distributions of W_i_ obtained for each individual experimental condition were assessed and used as the basis of the calculation of the un-weighted ensemble-average of W for the N experimental repeats:

$$\langle W\rangle =\frac{1}{N}\sum_{i=1}^{N}W_{i}$$ (2)

### MGL^+ve^ cell: MUC1-Tn/STn^+ve^ cell interaction assay

T47D-STn and T47D Simplecells were trypsinised and washed in PBS to form a single cell suspension before being stained with 10uM eFluor 670 (ebioscience) according to the manufacturer's instructions. K562, KG11 and immature and mature mo-DCs were washed in PBS before being stained with 10uM CFSE (ebioscience) according to the manufacturer's instructions. The cells were then incubated together at a 1:1 ratio at 4C for 2 hours in the presence or absence of calcium (0.5% BSA in PBS plus 5mM Ca^2+^ or 5mM EDTA respectively). After 4 hours, cell:cell binding was assessed by flow cytometry and the data was analysed using Cellquest software.

### Immunohistochemistry

#### Tissue

Forty formalin fixed paraffin embedded breast carcinomas were obtained from the King’s Health Partner’s Cancer Biobank, Guy’s Hospital. Ethical approval has been obtained for the use of tissue from this Bank in cancer research. Research Ethics approval was given by the Guy’s and St Thomas’ Research Ethics Committee, number 07/H0804/131 and extended by the East of England—Cambridge Research Ethics Committee. Tissue was collected with informed written consent unless collected prior to 30^th^ September 2006, before which the need for consent was waived by the review board. All cases were stained with haemotoxylin and eosin and reviewed by a breast pathologist. The study was performed under Guy’s & St Thomas’ Breast Tissue & Data Bank ethical approval (07/H0804/131). The type of carcinomas is described in S1 Table. 3μm sections were cut, dewaxed and stained with 10μg/ml of the monoclonal antibodies HMFG2, 5E5 [24] and 1B9 [24] or TKH2 as previously described [11]. Binding was visualised by the use of a biotinylated goat anti-mouse secondary antibody followed by streptavidin-HRP (DAKO). For double staining sections were incubated sequentially: Firstly sections were incubated with digoxigenated 1B9, washed and then incubated with ALEXA-488 labelled goat anti mouse, followed by incubation with biotinylated 5E5 and then washed and visualised with labelled ALEXA-568 Streptavidin (Molecular Probes).

#### Analysis of staining

The HRP sections were analysed and scored according to the percentage of cells within the tumour that were stained: 0 = no staining observed, >0≤1 = less than 25% of the tumour stained; >1≤2 = between 25% and 50% of the tumour stained; >2≤3 = between 51% and 75% of the tumour stained; >3≤4 = 76%-100% of the tumour stained.

#### In situ proximity ligation assay (PLA)

The proximity ligation assay kit (Duolink) was purchased from OLINK Biosciences (Uppsala, Sweden) and performed according to the manufacturer’s instructions. Briefly, after incubating sections with 10μg/ml of 5E5-biotin and 1B9-digoxigenin and washing, oligonucleotide-conjugated PLA secondary probes to biotin and digoxigenin were added and the sections incubated at 37°C for 2 hours. Two connector oligonucleotides were hybridised to the probe pairs for 15 minutes and then ligated together to form a circular DNA molecule. This was amplified through rolling circle amplification that can be detected through the hybridisation of fluorescently labelled complementary oligonucleotides.

## Results

### MUC1-Tn and STn expression by breast cancer

**Fig 3:**
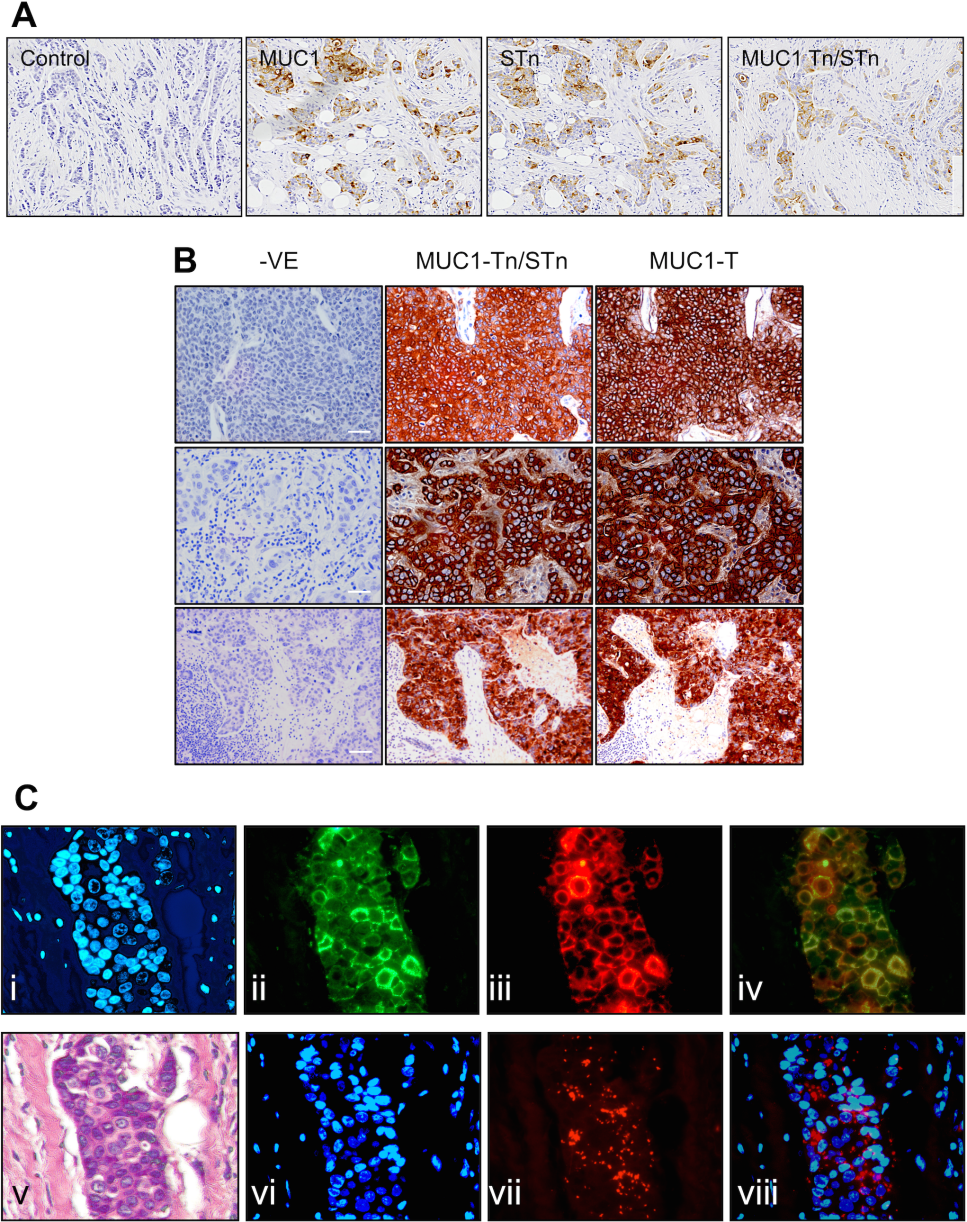
The glycans STn, Tn and T can be found in the same breast cancers in close proximity. Serial sections from formalin fixed paraffin embedded breast carcinomas were stained for MUC1, MUC1-Tn/STn, MUC1-T and the STn glycan using HMFG2, 5E5, 1B9 and TKH2 antibodies respectively.—VE or control = secondary antibody only. A, Expression of MUC1-Tn and STn by the same tumour, B. Three representative breast carcinomas showing expression of MUC1-Tn and MUC1-T. Scale bars = 100μM. C. Proximity ligation assay showing MUC1-Tn and MUC1-T are in close proximity. Formalin fixed paraffin embedded breast carcinomas were stained with i, DAPI to visualise the nuclei, ii, 1B9 (green) that recognizes MUC1-T; iii, 5E5 (red) that recognised MUC1-Tn. Co-localisation is seen by the yellow colour in iv. Proximity ligation assay (PLA) was performed, see Materials and Methods, vi DAPI staining to localise nuclei; vii PLA using 5E5 and 1B9; viii shows overlay of F and G. v shows H and E staining of the breast carcinoma.

The majority of breast cancers express the Tn antigen and around 25% express STn (see Fig 3A for examples of staining). To investigate the role of COSMC in Tn expression, 40 cases of primary breast cancer were stained with antibodies to MUC1 (HMFG2), MUC1-Tn/STn (5E5) and MUC1-T (1B9), see S1 Table for the results of the individual tumors. All but one expressed MUC1 and, of these, all gave positive staining with 5E5 demonstrating expression of MUC1-Tn/STn. All but two of the MUC1 expressing tumours stained with 1B9 demonstrating the presence of the T glycan on MUC1 in the same tumours that express Tn (see Fig 3B).

To determine if Tn and T were in close proximity, the proximity ligation assay was applied to sections of primary breast cancers using 5E5 coupled to biotin and 1B9 coupled to digoxigenin (see Materials and Methods). Initially, sections were sequentially stained with digoxigenin 1B9 (see Fig 3Cii) followed by biotin 5E5 (see Fig 3Ciii). Overlaying of the two images shows co-localisation of 5E5 and 1B9 staining within the same cells and to the same area within the cell. The PLA was then applied to the sections and as can be seen in Fig 3Cvii and viii a positive signal was obtained with the 5E5 and 1B9 antibodies indicating that not only can MUC1 expressing Tn and T be found within the same cell but that they are in very close proximity, less than 30nM apart, in all likelihood on the same molecule. This pattern of staining was demonstrated in three out of three individual breast carcinomas.

### MUC1 carrying Tn and STn binds to MGL

Recombinant MUC1 protein carrying Tn or STn was manufactured and purified as described in Material and Methods. The purity of MUC1 was confirmed by amino acid analysis, silver staining, the LAL endotoxin assay (data not shown) and by reactivity with the 5E5 antibody, which binds to Tn and STn but only when carried on MUC1 (S1 Fig) [24].

**Fig 4:**
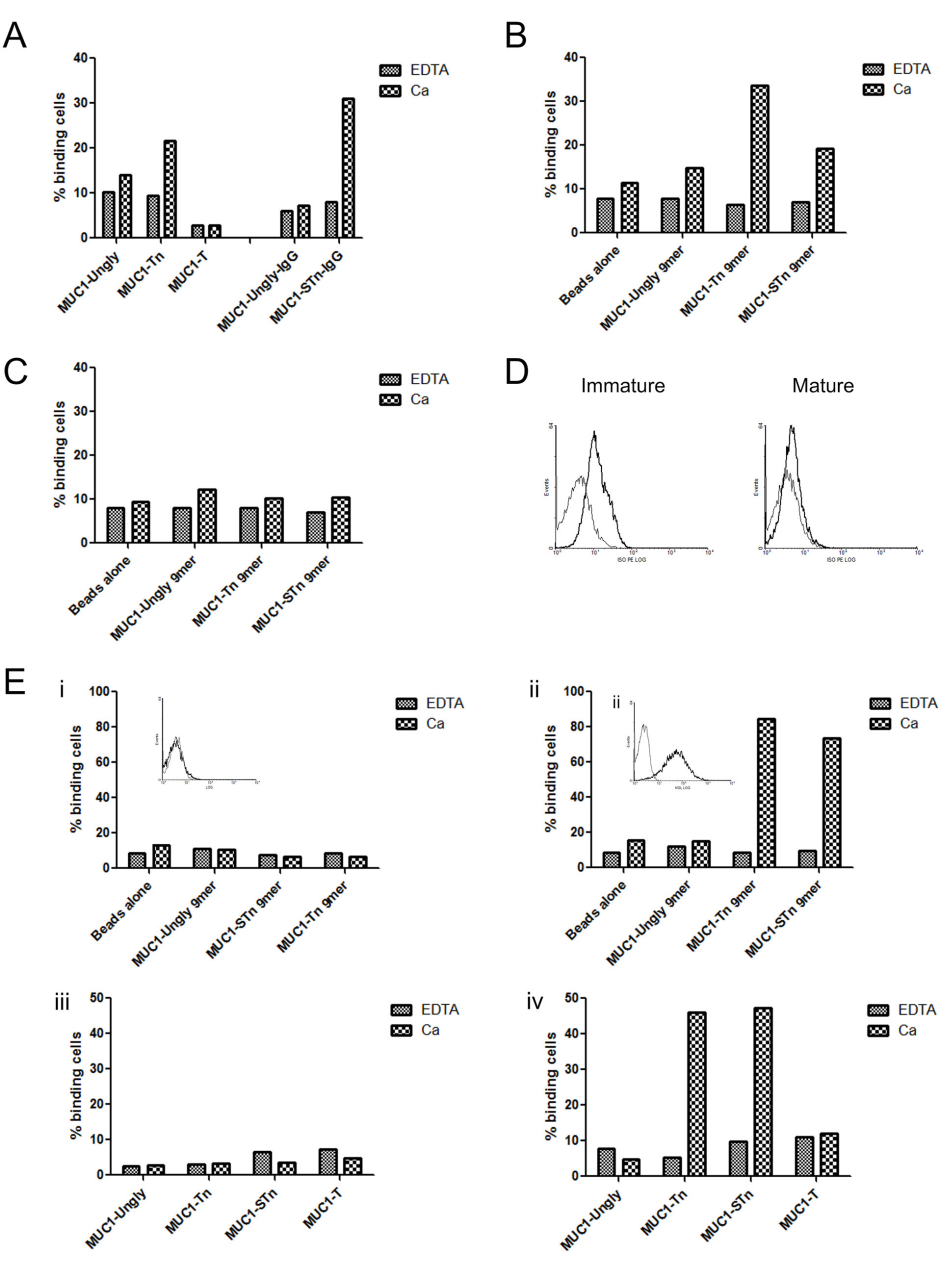
Cells expressing MGL bind to MUC1-STn glycopeptides and glycoproteins in a calcium dependant manner. Immature moDC were generated as described in materials and methods and incubated with fluorescent beads pre-coated with A, recombinant MUC1 glycoproteins; B, MUC1 glycopeptides. Cell binding was assessed by flow cytometry, with the % of binding cells shown. One experiment representative of three independent experiments is shown. C, MoDC were matured with LPS and the binding of MUC1 glycopeptides determined as above. D, MGL expression levels on immature and mature monocyte derived DCs, black; isotype control: bold black; anti-MGL binding. E, K562 (i,iii) and K562 transfected with MGL (ii,iv) were incubated with fluorescent beads pre-coated with recombinant MUC1 glycopeptides (i,ii) or recombinant MUC1 glycoproteins (iii,iv). Cell binding was assessed by flow cytometry, with the % of binding cells shown. One experiment representative of two independent experiments is shown. Inserts show the expression of MGL for K562 cells and K562 cells transfected with MGL, black; isotype control: bold black; anti-MGL binding.

Fig 4A shows that both the Tn and STn recombinant MUC1 glycoforms bind to immature dendritic cells whereas MUC1 carrying the core 1 (Galβ1,3GalNAc, the T antigen) or unglycosylated MUC1 show no binding greater than observed in the absence of calcium. Binding was also observed with a 60 mer peptide comprised of 3 tandem repeats of MUC1 carrying 9 Tn or STn glycans (Fig 4B). However, the binding of MUC1-Tn and STn to mature DCs was not observed (Fig 4C). MGL is expressed by immature dendritic cells, and has been shown to bind MUC1-Tn [18], however the expression of MGL is reduced or lost when the DCs mature (see Fig 4D) which correlates with the binding pattern of MUC1-Tn and MUC-STn. To confirm MUC1-Tn and MUC1-STn was indeed binding to MGL we measured the binding of these glycoforms to K562 cells transfected with MGL (KG11). Fig 4E shows that both MUC1-Tn and MUC1-STn glycopeptides and glycoproteins bind to the K562 transfected with MGL (KG11) but not the untransfected parental cells.

**Fig 5:**
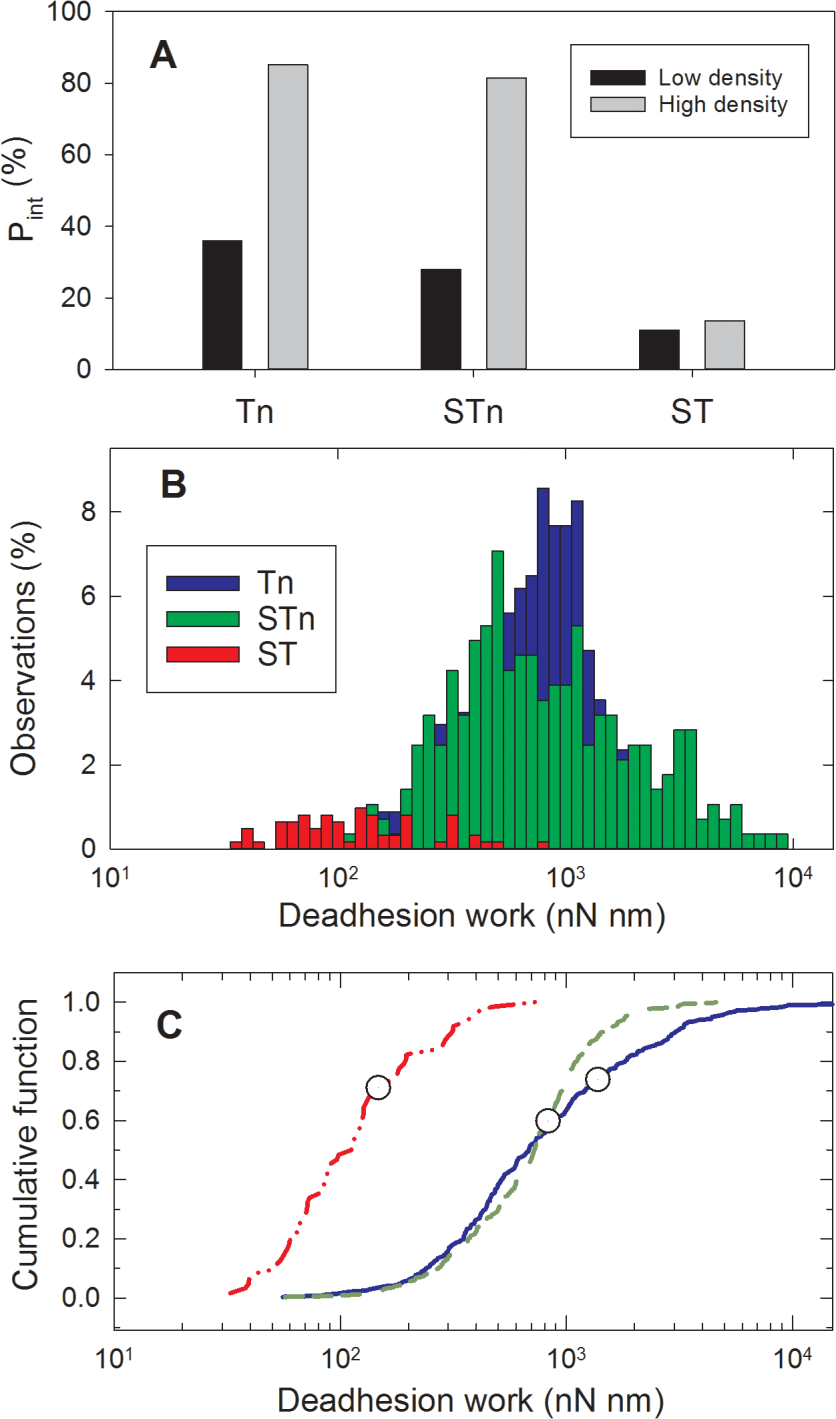
MUC1-Tn and STn show a similar and density dependent deadhesion work when allowed to interact with MGL, whereas MUC1-ST show a low and density independent deadhesion work. A: Fraction of total number of AFM force—distance curves displaying signatures of interaction (P_int_). The P_int_ values determined based on curves obtained using AFM tips functionalised with a low or a high density of glycans are presented separately. B: Distribution of work performed on the MUC1-Tn-MGL (blue), MUC1-STn–MGL (green) and MUC1-ST–MGL (red) molecular interactions in forced unbinding experiments. performed using an AFM tip retraction speed equal to 2 μm/s. The histogram distributions are based on the following number of observations: MUC1-Tn: 339, MUC1-STn: 283 and MUC1-ST: 62. C: Cumulative fraction of the distributions presented in panel B of work performed on the MUC1–MGL molecular pairs during the forced unbinding. The colour codes are the same as in B. The symbols represent the mean de-adhesion work performed on the MUC1-MGL molecular pairs (Eq (2)) and are located at corresponding percentiles in the cumulative distributions.

To gain further insight into the characteristics of the recombinant Tn- and STn-MUC1 glycoforms interacting with MGL, atomic force microscopy (AFM) was used (see Fig 2). The full-length recombinant MGL used in these experiments has been shown by the manufacturer R and D Systems, to form trimers (R and D Systems, personal communication) and maintained this structure during the AFM experiments. This clustering of the receptor reflects the trimeric structure observed in the cell membrane and so is representative of the *in vivo* situation [30]. The interactions were investigated both at low and at high density of MUC1 immobilised onto the AFM tip. For the experimental series obtained using AFM tips with low density of the immobilised MUC1 molecules, a relatively small fraction of the curves obtained displayed signatures of forced bond rupture. For MUC1-Tn–MGL interactions 36% of the curves contained such signatures, for MUC1-STn–MGL this fraction was 28% and for MUC1-ST—MGL the fraction decreased to 11% (see Fig 5A). In the case of MUC1-Tn or MUC1-STn, increasing the density on the AFM tip resulted in an increased fraction of AFM curves containing evidence of interaction (P_int_), to 85 and 81 respectively (see Fig 5A). The correlation between the density of the active molecules and P_int_ indicates that for these samples the observed rupture events are due to the MGL—MUC1 interaction. MUC1 carrying the ST O-glycan behaved differently; an insignificant change in P_int_ was observed (from 11 to 14%) upon increasing the density of MUC1-ST on the AFM tip (see Fig 5A).

The distribution of the de-adhesion work for the MUC1-Tn and MUC1-STn-MGL interactions is displaced towards larger de-adhesion work compared to the MUC1-ST-MGL interaction (see Fig 5B). This reflects a higher number and/or strength of the total intermolecular rupture events in AFM curves where signatures of interaction were observed. The data are based on 4667 AFM force curves. Due to the difference in the likelihood of interaction (see Fig 5A), the histogram is for MUC1-ST based on fewer AFM force distance curves than the histograms for MUC1-Tn- and-STn. The cumulative distributions of the work performed on the MUC1—MGL (Eq (1)) are presented in Fig 5C on a logarithmic scale of W. The meanW, <W> (Eq (2)), depicted by a circular symbols in Fig 5C, is for the MUC1-STn–MGL interaction 1380 nN nm, and is reduced to 830 nN nm for the MUC1-Tn-MGL interaction, and is further reduced to 147 nN nm for the MUC1-ST-MGL interaction. This difference in the average value of the de-adhesion work indicates a stronger tendency of MUC1-Tn and STn to form adhesive interactions with the MGL functionalized surface compared to MUC1-ST.

The weak correlation between the amount of interaction and the density of the MUC1-ST indicates that the adhesion events observed for this sample is not due to a specific MUC1-ST–MGL molecular bond but to unspecific adhesion between AFM tips and mica surface. Such unspecific adhesive interactions were previously quantified in our laboratory [31]. The possible existence of unspecific anchoring events was therefore quantified in an experimental series where the MUC1 functionalized AFM tips where allowed to approach surfaces functionalized with either silane or glutardealdehyde terminated surfaces. Adhesion was indeed observed at the contact point between the tip and these mica surfaces. This shows that in cases of incomplete coverage of the surfaces with functional molecules under study (MUC1 and MGL proteins) signatures of unspecific adhesion can be expected, and these may account the adhesive events observed for the MUC1-ST sample.

#### Competition studies

**Fig 6:**
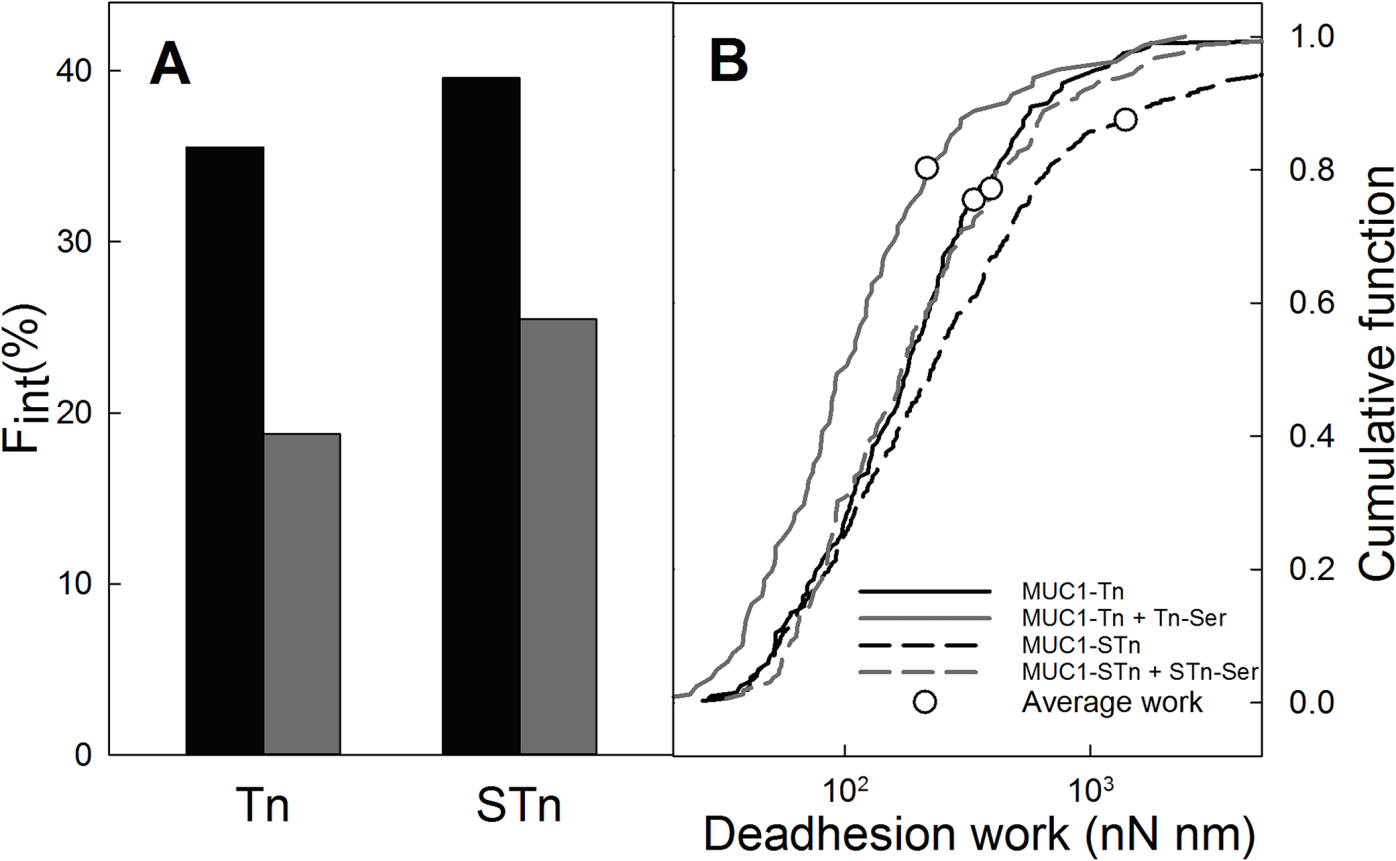
Probability for rupture of MUC1-Tn and MUC1-STn MGL interactions with MGL prior to and after the addition of free Tn-Ser or STn-Ser, respectively, to the buffer solution. A: Fraction of total number of AFM force—distance curves displaying signatures of intermolecular rupture events (F_int_), prior to (black) and after (grey) the addition of Tn-Ser or STn-Ser to a concentration equal to 0.5 mg/ml. B: Cumulative fraction of work performed during the forced unbinding of MUC1-Tn (continuous lines) and MUC1-Tn (dotted lines) from MGL. The analysis is performed based on the fraction of the AFM force-distance curves where interaction events were observed. The total deadhesion work determined both prior to (black lines) and after (grey lines) adding Tn-Ser or STn-Ser are displayed. The symbols represent the mean de-adhesion work performed on the MUC1—MGL molecular pairs (Eq (2)) and are located at corresponding percentiles in the cumulative distributions.

To confirm the specificity of the interactions, the effect of adding of adding Tn-Ser to MUC1-Tn and STn-Ser to MUC1-STn interactions with MGL was studied. Upon addition of Tn-Ser or STn-Ser to the buffer, a shift towards reduced de-adhesion work was observed both for the MUC1-Tn and the MUC1-STn systems. Fig 6 presents the de-adhesion work performed when separating the MUC1-Tn or MUC1-STn functionalized AFM tips from MGL-functionalized surfaces prior to as well as after the addition of Tn-Ser or STn-Ser to the buffer solution to a concentration equal to 0.5 mg/ml. The meanW, <W> (Eq (2)), depicted as a circular symbols in Fig 6, is for the MUC1-STn–MGL interaction reduced from 1397 nN nm to 394 nN nm, a 72% decrease. For the MUC1-Tn-MGL interaction, a decrease of 36% is observed, from <W> equal to 336 nN nm to 216 nN nm. At this point it cannot be ruled out that the difference in the percentage decrease observed between the two samples might be related to differences in molecular density on the surfaces used when obtaining these experimental series and further investigations should be performed before drawing conclusions based on this difference. Experiments performed by adding free GalNAc to the buffer solution when quantifying MUC1-Tn and MUC1-STn binding to MGL revealed that the addition of free GalNAc to both systems resulted in decreased deadhesion work of 77% for 0.02mg/ml GalNAc (data not shown). These data are in accordance with the previous data affirming that the MUC1-Tn-MGL interaction is out competed by the addition of GalNAc [18,32]. The reduced probability for interaction observed upon the addition of Tn-Ser, STn-Ser or GalNAc to the buffer solution adds further evidence to the conclusion that the force jumps observed in the AFM force distance curves when allowing MUC1-Tn or MUC1-STn functionalized AFM tips to interact with MGL surfaces are indeed due to the specific interaction between these MUC1 molecules and MGL. Moreover, binding of MUC1-STn to MGL can be out competed by GalNAc.

### MUC1-STn and MUC1-Tn expressing cell lines can bind to K562 cells transfected with MGL

**Fig 7:**
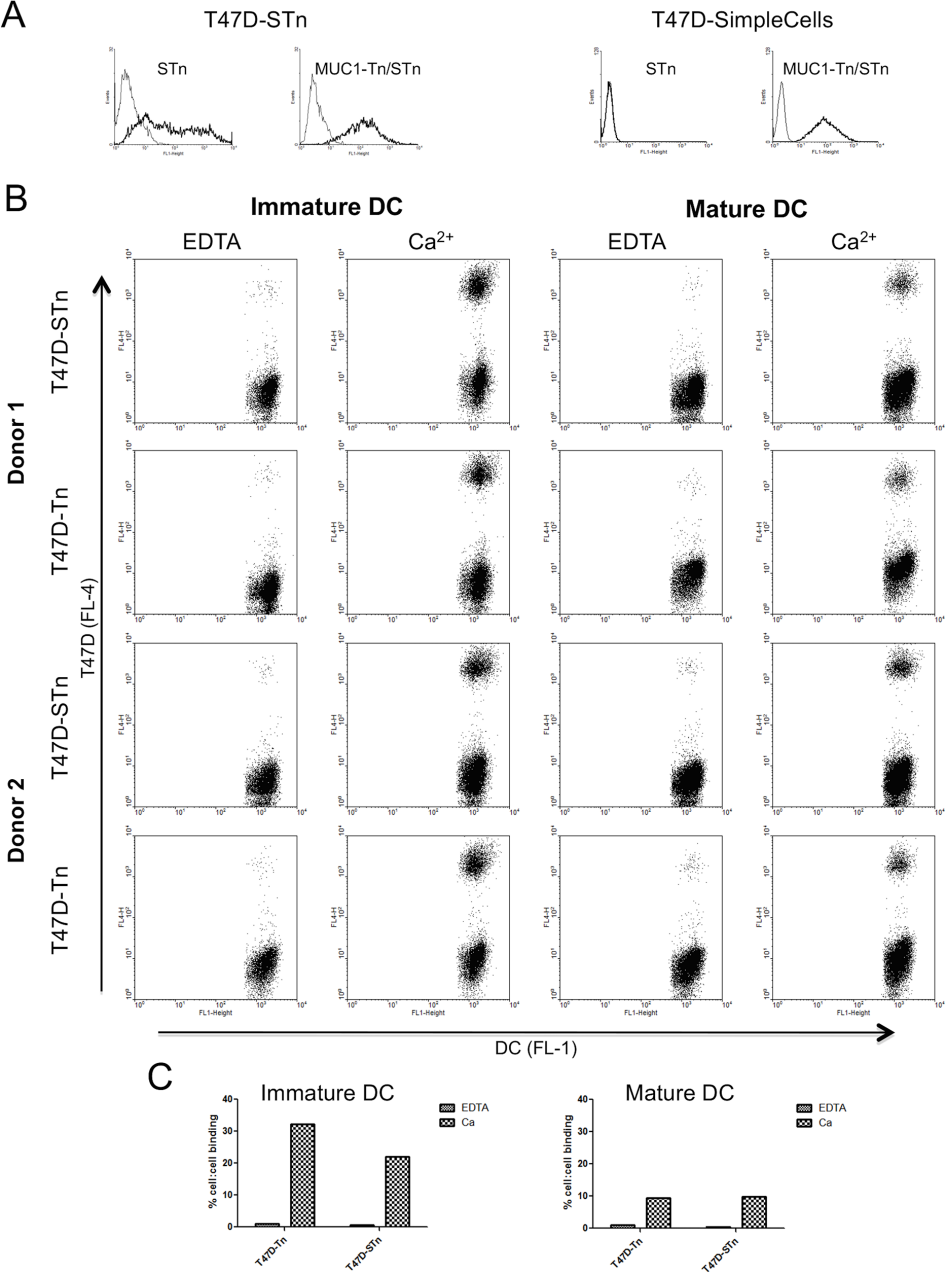
T47D breast carcinoma cells expressing either STn or Tn, can bind to immature mo-DC in a calcium dependant manner. A, T47D-STn and T47D-Tn (SimpleCells) were stained with antibodies to STn (TKH2) or MUC1-Tn/STn (5E5), black; isotype control: bold black antibody binding. B, dot-plots illustrating the interaction between T47D cells and immature or mature mo-DCs in the presence or absence of calcium. T47D cells and moDCs were stained with dyes (eFluor 670 and CFSE respectively) before being incubated on ice together for 4h. Cell:cell interaction was measured by flow cytometry as double positives. Cells were gated using CFSE and FSC. C. Graphic representation of figure B.

**Fig 8:**
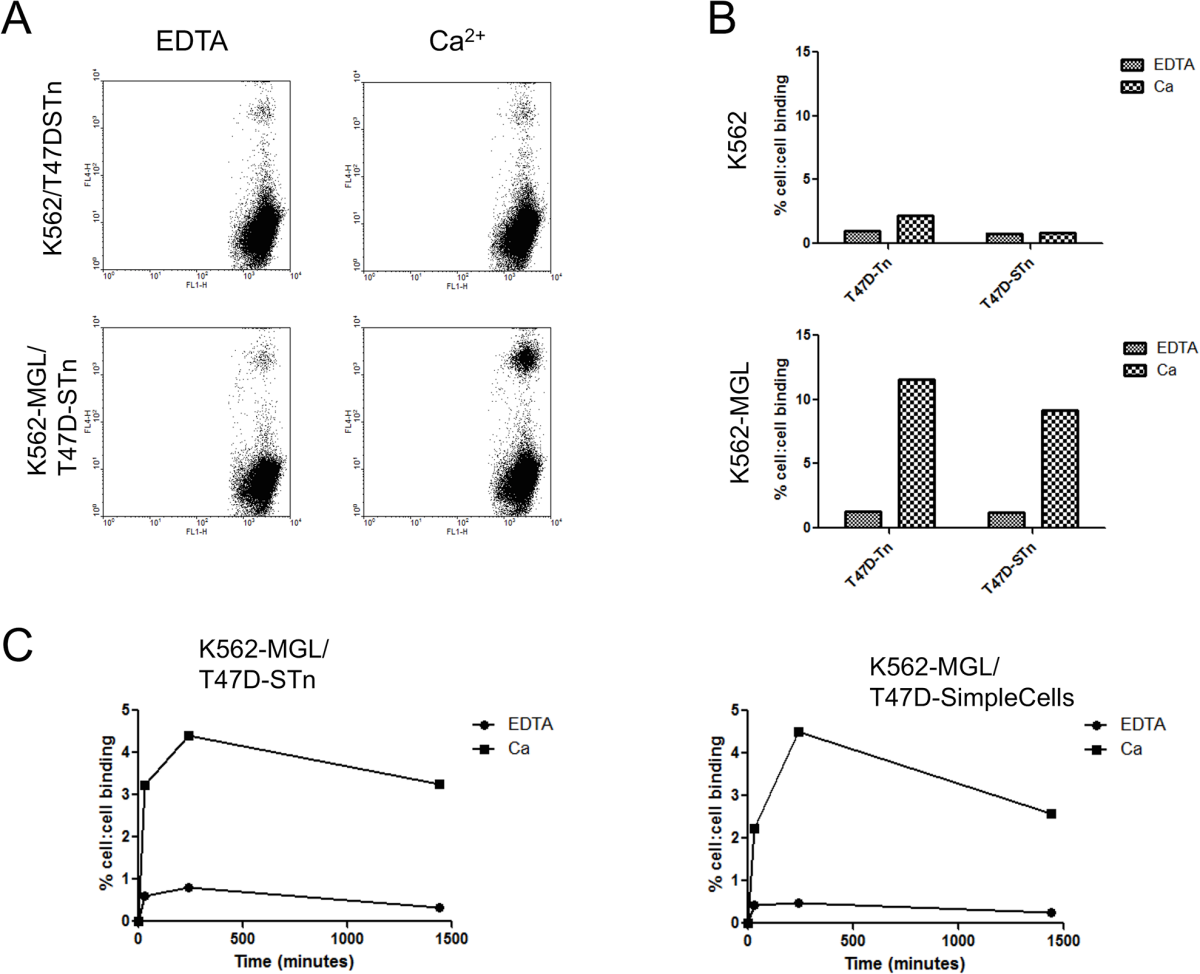
T47D breast carcinoma cells expressing either STn or Tn, can bind to K562-MGL cells in a calcium and time dependant manner. A, dot-plots illustrating the interaction between T47D-STn cells and K562 or K562-MGL cells in the presence or absence of calcium. T47D-STn and K562 cells were stained with dyes (eFluor 670 and CFSE respectively) before being incubated on ice together for 4h. Cell:cell interaction was measured by flow cytometry as double positives. Cells were gated using CFSE and FSC. B, graphic representation of figure A with the addition of T47D-Tn binding to K562-MGL. C, measuring this interaction at different time points reveals the peak interaction occurs between 4 and 24h for both T47D-Tn and T47D-STn cells.

Having demonstrated that MUC1-Tn and MUC1-STn show a similar capacity to interact with MGL, it was important to show an interaction between cell surface MUC1-STn and MGL. Although *in vivo* around 25% of breast carcinomas express STn, few cell lines express this glycan. We therefore made use of T47D cells transfected with ST6GalNAc-I, which results in the expression of STn on the cell surface [14], see Fig 7. In addition, to ensure surface expression of Tn we used T47D SimpleCells that had *COSMC* knocked-out via gene editing [33] to investigate binding to Tn (see Fig 7). Using flow cytometry we showed that T47D carried Tn or STn on the cell surface (Fig 7A) and they interacted with monocyte derived immature dendritic cells and to a much lesser degree with mature dendritic cells, in a calcium dependent manner, see Fig 7B and 7C. However, dendritic cells express a number of C-type lectins so we cannot rule out the fact that the binding observed could be a composite of interactions with other lectins. We therefore investigated the binding of T47D-STn expressing cells to K562 cells and K562 cells transfected with MGL. As shown in Fig 8, T47D-STn cells bound to K562 cells expressing MGL in a calcium dependent manner but not to the parental K562 cells. It was also shown that this interaction was time dependant, with optimum binding at 4°C occurring at approximately 4 hours. Thus breast cancer cells carrying either Tn or STn on their surface can interact with MGL expressing cells.

## Discussion

Changes in glycosylation are common events in cancer and can lead to aberrant interactions of glycoproteins expressed by the tumour cell with glycan binding proteins, including C-type lectins expressed by normal cells. In the case of binding to lectins on immune cells this may induce the secretion of inflammatory mediators that are believed to drive tumour growth and progression or may induce the uptake of soluble glycoproteins leading to antigen presentation [6,1].

Breast cancer can carry the truncated glycans Tn and sialyl-Tn (STn). One mechanism that may result in O-linked glycoproteins carrying these truncated glycans is the inactivation or lack of expression of COSMC. As mucin O-glycans are synthesised by the sequential addition of individual sugars, inactivation of COSMC, resulting in a non-functional T synthase, blocks further chain elongation so that O-glycosylation is truncated to Tn or STn if ST6GalNAc-I is active, and T glycoforms should not be detected [15]. In the results described here we have shown that in breast cancers expressing Tn, not only can T be expressed in the same area of the tumour, as demonstrated by staining of sequential sections of 39 breast carcinomas, but double staining shows that these glycans can be found in the same cells. Importantly, the application of the Proximity Ligation Assay demonstrated that Tn and T are in very close proximity, suggesting they are carried on the same molecule or molecules in very close contact. This suggests that *COSMC* is still active in these breast cancer cells. Moreover in the large METABRIC study, expression of *COSMC* was in fact increased in breast cancer compared to normal (1.5 fold, p = 5.15E-6) [34] suggesting that unlike that observed in pancreatic cancer [16], silencing of *COSMC* expression by DNA methylation may not play a role in dictating the expression of Tn and STn in breast cancer, although this can only be confirmed by determining the T synthase activity.

When glycans are carried on MUC1 they can be repeated hundreds of times. Depending on the allele, the extracellular domain of MUC1 consists mainly of 25–150 tandem repeats of 20 amino acids. Each repeat carries five O-linked glycosylation sites, thus glycans can potentially be repeated 125–625 times on each molecule allowing engagement and crosslinking of relevant lectins. To begin to understand the implications of these truncated glycans being carried on MUC1 we focused our studies on MGL binding. Human MGL was thought to exclusively recognise GalNAc [35,36], however recently MGL has been shown to bind free STn [30,37]. Although the binding of glycoprotein carrying Tn has been investigated [18,19] the binding of STn carrying proteins to MGL has not been robustly studied. To investigate this area we produced highly purified recombinant human MUC1 glycoproteins and MUC1 glycopeptides carrying either Tn or STn glycans and showed that MUC1 carrying Tn and STn could bind MGL expressed by immature monocyte-derived dendritic cells and by K562 transfected with MGL. Although these flow cytometry based techniques appeared to show similar binding, this technique cannot be used to quantitate affinity, therefore we used AFM to measure the relative deadhesion work. These studies showed that MUC1 carrying Tn or STn behaved in a similar way. When comparing results obtained for AFM tips obtained using identical experimental conditions, these two samples showed a similar and density dependent deadhesion work, which was also reduced upon the addition of the competing compounds Tn-Ser, STn-Ser or free GalNAc, demonstrating the specificity of the observed interactions. This was in contrast with the observations made for MUC1-ST, which showed a low and density independent deadhesion work. Although our data showing binding of MUC1-STn to MGL agrees with published data showing binding of the STn hapten to MGL, it differs in that using NMR and SPR, Tn and STn were shown to bind MGL with differing affinities [37]. This deviation might be caused by the inherent properties of the direct forced unbinding assay, which requires immobilization and investigation under non-equilibrium conditions, differing from that of the NMR based characterization.

Lectins expressed by immune cells have been thought to be involved in the recognition of PAMPs (pathogen associated patterns) although it is now clear that they can also bind host glycans. Indeed lectins are involved in the recognition of DAMPs (danger associated molecular patterns) released during tissue damage [38] and it has been proposed that lectins may also recognise SAMPs (self-associated molecular patterns) and RAMPs (resolution-associated molecular patterns) to maintain or re-establish the baseline of non-activation of the innate immune system [39]. Moreover, MGL is involved in deactivating the adaptive immune response as it binds to CD45 expressing activated T cells carrying Tn resulting in a reduction of T cell proliferation and T cell death [40]. Engagement of MGL has also been shown to inhibit the migration of dendritic cells and it is only upon maturation when MGL expression is down-regulated that the cells migrate out of the lymphoid organs and encounter antigen [41].

MGL has been reported to be a marker of alternative activation in murine models of allergy and parasitic infection, with IL-4 and IL-13 being shown to be crucial for its induction and maintenance [42]. Indeed, IL-4 and IL-13 are now considered key players in alternative macrophage generation, including tumour associated macrophages (TAMs) [43]. Moreover, in human studies MGL is more highly expressed in alternatively activated M2 than M1 macrophages and in TAMs isolated from ovarian carcinomas [44]. Thus antigens taken up via MGL will be presented in an alternative fashion in the tumour microenvironment. Although binding of MUC1-Tn to MGL has been shown to facilitate the uptake of MUC1 and antigen presentation [18], which induces signalling through the MAPK ERK pathway [45,46], co-commitment of TLR triggering was required for IL-10 and TNF**α** secretion [45,46].

Whilst STn is a terminal glycan, the expression of surface terminal GalNAc structures is unusual in breast cancer as it must be a result of incomplete O-linked glycosylation as our data rules out the cause being lack of COSMC function. Indeed the Tn glycan is mostly intracellular and not frequently found on the surface of carcinomas [20]. Thus the binding of MUC1 carrying STn to MGL may be more physiologically relevant than the binding of MUC1 carrying Tn. Tumour associated STn is associated with poor prognosis and resistance to chemotherapy in breast carcinomas [12], inhibition of DC maturation [47], DC apoptosis [48] and inhibition of NK activity [49]. As data suggest that engagement of MGL in the absence of TLR triggering may lead to anergy, the binding of MUC1-STn to MGL may be in part responsible for some of the characteristics of STn expressing tumours.

## Supporting Information

S1 Fig MUC1-Tn and MUC-STn react with 5E5 but with different affinities. 1μg of recombinant MUC1-Tn or MUC1-STn was coated onto each well of a 9well dish. After washing 5E5 was added and the binding visualized with rabbit anti-mouse IgG peroxidase conjugated.
(PPTX)

S1 Table Breast carcinoma express MUC1 carrying Tn and/or T and ST. Results of the IHC of the individual tumours.
(DOCX)
