# Supplementary material for: The Breast Cancer-Associated Glycoforms of MUC1, MUC1-Tn and sialyl-Tn, Are Expressed in COSMC Wild-Type Cells and Bind the C-Type Lectin MGL
Source: PLoS One. 2015 May 7;10(5):e0125994. doi: 10.1371/journal.pone.0125994 (PMC4423978; doi:10.1371/journal.pone.0125994)
Supplement: S1 Fig — 1μg of recombinant MUC1-Tn or MUC1-STn was coated onto each well of a 9well dish. After washing 5E5 was added and the binding visualized with rabbit anti-mouse IgG peroxidase conjugated. (PPTX) [file pone.0125994.s001.pptx]

## Slide 1
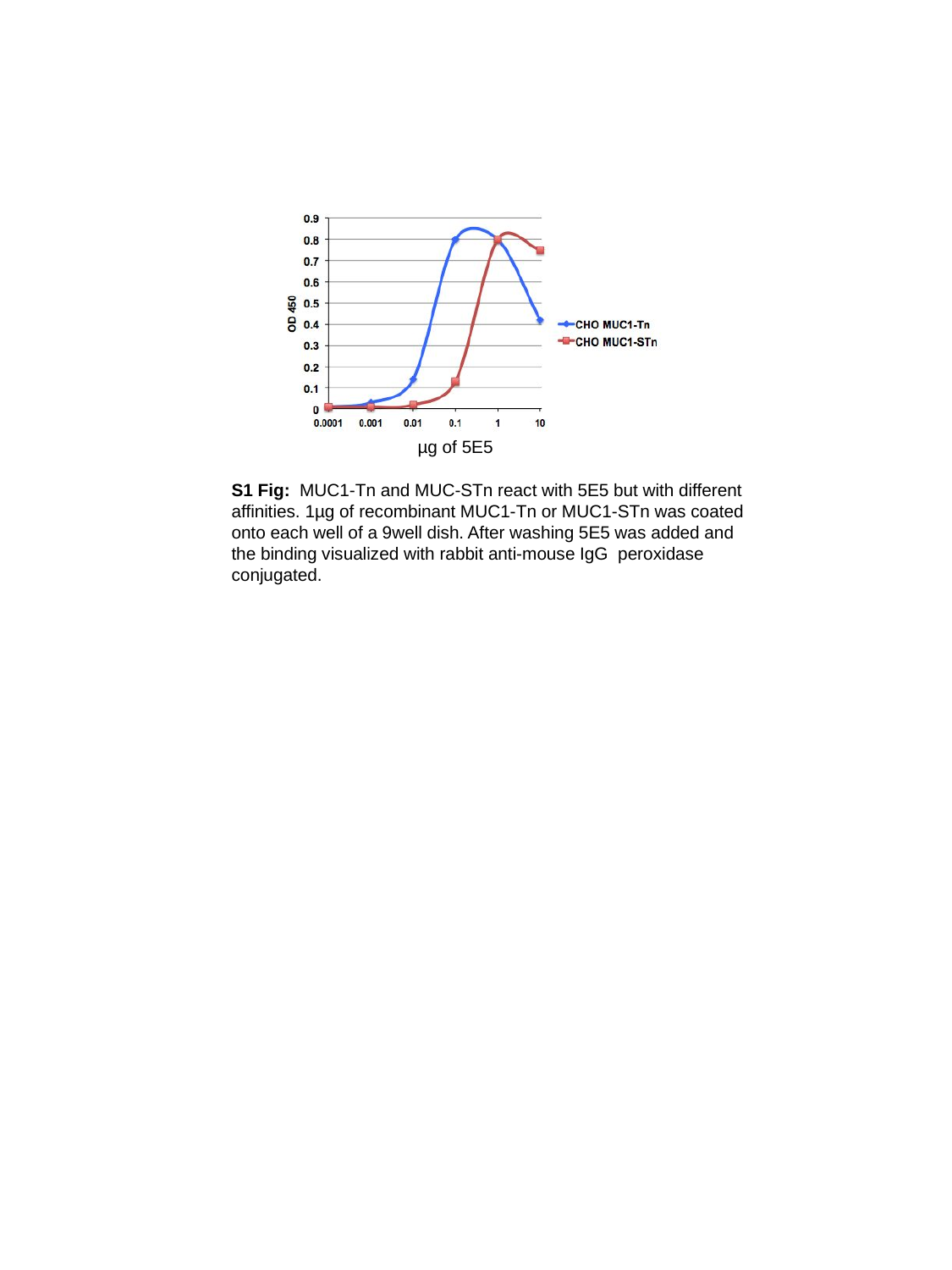

µg of 5E5
S1 Fig: MUC1-Tn and MUC-STn react with 5E5 but with different affinities. 1µg of recombinant MUC1-Tn or MUC1-STn was coated onto each well of a 9well dish. After washing 5E5 was added and the binding visualized with rabbit anti-mouse IgG peroxidase conjugated.
