# Supplementary material for: The Breast Cancer-Associated Glycoforms of MUC1, MUC1-Tn and sialyl-Tn, Are Expressed in COSMC Wild-Type Cells and Bind the C-Type Lectin MGL
Source: PLoS One. 2015 May 7;10(5):e0125994. doi: 10.1371/journal.pone.0125994 (PMC4423978; doi:10.1371/journal.pone.0125994)
Supplement: S1 Table — Results of the IHC of the individual tumours. (DOCX) [file pone.0125994.s002.docx]

**S1 Table: Breast carcinoma express MUC1 carrying Tn and/or T and ST**

| Tumour type | Grade | Antibody Staining Score | | | |
| --- | --- | --- | --- | --- | --- |
|  |  | HMFG2  (MUC1) | SE5  (Tn-MUC1) | IB9  (T-MUC1) | IB9  (after neuraminidase treatment) |
| Ductal | 3 | 3 | 3 | 1 | 2 |
| Ductal | 3 | 3 | 2 | 1 | 2 |
| Ductal | 3 | 3 | 2 | 0.5 | 2 |
| Ductal | 3 | 0 | 0 | 0 | 0 |
| Ductal | 2 | 2 | 2 | 0.5 | 1 |
| Ductal | 3 | 4 | 3 | 2 | 3 |
| Ductal | 3 | 4 | 3 | 1 | 2 |
| Ductal | 3 | 2 | 3 | 1 | 2 |
| Ductal | 2 | 4 | 3 | 2 | 2.5 |
| Ductal | 3 | 3 | 3 | 1 | 3 |
| Ductal | 2 | 4 | 3 | 2 | 2 |
| Ductal | 3 | 1 | 1 | 0.1 | 0.5 |
| Ductal | 3 | 2 | 2 | 0 | 0.5 |
| Ductal | 2 | 4 | 4 | 3 | 4 |
| Ductal | 2 | 4 | 4 | 2 | 3 |
| Ductal | 3 | 4 | 4 | 2 | 4 |
| Ductal | 3 | 3.5 | 3 | 2 | 2.5 |
| Ductal | 1 | 4 | 4 | 1 | 3 |
| Ductal | 3 | 3 | 3 | 1 | 2 |
| Ductal | 3 | 4 | 3 | 0 | 3 |
| Ductal | 3 | 4 | 4 | 2 | 3 |
| Ductal | 3 | 4 | 4 | 3 | 4 |
| Ductal | 3 | 4 | 4 | 4 | 4 |
| Ductal | 1 | 4 | 4 | 3 | 3.5 |
| Ductal | 3 | 4 | 3.5 | 2 | 2 |
| Ductal | 3 | 4 | 4 | 3 | 3 |
| Ductal | 3 | 4 | 4 | 3 | 3.5 |
| Ductal | 1 | 3 | 2.5 | 2 | 2 |
| Ductal | 1 | 4 | 4 | 1.5 | 2 |
| Ductal | 1 | 2 | 1.5 | 0.5 | 1 |
| Ductal | 2 | 4 | 1.5 | 1 | 1 |
| Ductal | 3 | 4 | 3 | 1.5 | 2.5 |
| Ductal | 3 | 4 | 3.5 | 1 | 3 |
| Ductal | 1 | 3 | 2 | 0.5 | 2.5 |
| Ductal | 3 | 4 | 3.5 | 2.5 | 3 |
| Ductal tubular | 1 | 2 | 2 | 1 | 2 |
| Lobular | 2 | 4 | 3.5 | 2 | 3 |
| Lobular | 2 | 4 | 4 | 2.5 | 3 |
| Lobular | 2 | 4 | 3.5 | 2.5 | 3 |
| Lobular | 1 | 4 | 3.5 | 2.5 | 3 |
